# ERP Staff versus AI recruitment with employment real-time big data

**DOI:** 10.1007/s44163-022-00037-1

**Published:** 2022-10-31

**Authors:** Kenneth David Strang, Zhaohao Sun

**Affiliations:** 1 W3-Research, Data Analytics, St. Thomas, VI 00802 USA; 2 Business Research, RMIT University, North Sydney, NSW 2062 Australia; 3 Department of Business Studies, PNG University of Technology, Private Mail Bag, Lae, 411 Morobe Papua New Guinea

**Keywords:** Human resource management (HRM), Big data, Natural language processing (NLP), Artificial intelligence (AI), Machine learning (ML), Z-Test, ANOVA, Enterprise resources planning (ERP)

## Abstract

The purpose of this study was to evaluate the effectiveness of using natural language processing (NLP) artificial intelligence (AI) in enterprise resources planning (ERP) to identify specialized job candidates in real-time big data—globally across the internet. The central problem was that companies using traditional methods for recruiting remote specialists were missing good candidates because the skilled employees may not be looking for a job yet they may be receptive to an offer. The auxiliary problem was too much data on the internet for human resources management (HRM) staff to make sense of to find the best-fitting candidate. Thus, the research question was: could NLP AI identify good candidates for ERP remote specialist jobs using internet real-time big data? Job criteria were developed using machine learning to identify key skills from existing staff in a case study company. The skills were transformed into ERP remote specialists hiring criteria. The NLP AI software was activated to find the best candidate. The HRM staff at the case study company evaluated the effectiveness of the candidate selected by the NLP AI. The case study company set 70% as the acceptable mean evaluation score. ANOVA was used to determine if HRM staff agreed about their evaluation scores. A Z-test was used to determine if the NLP AI was faster than the mean time needed for HRM to select ERP candidates. The results were that the NLP AI outperformed the humans by a factor of almost 8 h. All HRM staff agreed that the NLP AI was effective in selecting a candidate to match the hiring criteria. The proposed approach might facilitate the research and development of big data, data analytics, NLP AI, and HRM process improvement.

## Introduction

The research question investigated in the current study was whether natural language processing (NLP) algorithms in an Artificial Intelligence (AI) program could be effective for recruiting enterprise resource planning (ERP) candidates from internet real-time big data. The underlying problem was that more jobs are transitioning to remote which opens the job pool to qualified candidates around the world. There are too much candidate big data on the internet for human resources management (HRM) staff to search. AI programs, particularly NLP algorithms, are capable of searching social media, job boards, and just about any site on the internet, to locate candidates, based on hiring criteria. The issue for the current study was whether an NLP AI could perform at least as well as a human for selecting a candidate who is a good match with the hiring criteria. The term ‘perform at least as well’ could mean performing faster and more objectively, with less discrimination. Recruiting is a strategic and critical activity, among many others, performed within an organization’s ERP function [1]. The recruiting function has become more digital and virtual due to new technology and the overall risk avoidance sentiments carried over from the COVID-19 pandemic [2]. Therefore, AI has been used in some ERP functions, to avoid travel, and to process high volumes of big data [3, 4]. The authors propose that NLP AI could be used in HRM.

Most of the recent literature reported that some types of AI were beneficial for ERP functions including performing repetitive tasks [5–7]. However, AI programs caused many ERP problems according to some researchers [8–11]. Amazon’s ‘secret hiring AI’ used during 2014–2017 was found to bias job candidates if the applications contained a woman’s salutation or name, and excluded certain colleges [9]. A BBC journalist researched and experimented with recruiting AI software including Pymetrics, Textico, and HireVue, asserting women and people of color were being overlooked by AI algorithms—including herself [12]. MIT researchers uploaded a fake job to My Interview and Curious Thing recruiting AI software then they applied it as an experiment—both programs failed, for example, by scoring a candidate high for English proficiency when she spoke only in German [13]. Thus, some types of AI algorithms created significant problems in the ERP function—discrimination is a serious legal problem in developed countries. It was unclear if NLP AI was tested in HRM.

Despite some problematic implementations of AI in ERP for recruiting, there was a counterargument in the literature that AI could be beneficial [6]. In a hiring experiment conducted by Bertrand and Mullainathan [14] where they used fake resumes to apply for jobs in two highly-populated US cities, they found that there was significant racial discrimination by humans even at companies that had claimed to be equal opportunity employers eligible for favorable tax and other government considerations. In a meta-analysis, Fraij and Laszlo [5] concluded that AI was beneficial for recruiting such as automating time-consuming processes like screening job applications and providing HRM staff with more quality time to focus on strategic activities. Other researchers claimed that AI was valuable and much needed to automate some of the ERP labor-intensive recruiting processes, although careful human oversight was needed [15, 16].

The above rationale, grounded in the literature, led to the research question (RQ): could NLP AI identify good candidates for ERP remote specialist jobs from internet real-time big data? A scientific study experiment was designed to answer the RQ at a selected company, using integrated methods. Pragmatic approaches were used to overcome the complexity of comparing NLP AI effectiveness in recruiting remote ERP job specialists from high-volume and high-velocity internet-based real-time big data.

The rest of this paper is organized as follows: Sect. 2 provides a literature review for this research and presents big data derived small data approach. Section 3 provides the research approach of this research. Section 4 explores the experiment, participants, and datasets for our research. Section 5 discusses the research results. Section 6 points out the limitations and future research directions. The last section ends this paper with some concluding remarks and recommendations for future research.

## Literature Review

This section provides a background on big data, big data analytics, machine learning, data mining, AI, NLP, data science, and ERP HRM as related to the current study.

### Big data and analytical approach

Big data is considered a frontier for research and development in many disciplines since 2012 [2, 17]. Big data is a transforming science that has impacted engineering, technology, medicine, healthcare, finance, business and management, education, and ultimately society—due to the emergence of big data analytics [18]. The big v’s refer to the five distinguishing characteristics of big data: big volume, big velocity, big variety, big veracity, and big value [19, 20]. Other researchers have added more features to help describe big data. Therefore, big data should have all the basic five characteristics to be considered big data, although some scholars argue that it is enough to meet only one of the five characteristics since each one is beyond analysis with most contemporary methods of statistical software [21, 22].

Big data analytics is a science with integrated technology for organizing big data, analyzing and discovering knowledge, patterns, and intelligence from big data, as well as visualizing and reporting the discovered knowledge and insights for assisting decision-making [22]. The main components of big analytics include big data descriptive analytics, predictive analytics, and prescriptive analytics [22], which correspondingly address the three kinds of questions of big data; when and what occurred? What will occur? What is the best answer or choice under uncertainty? All these questions are often encountered in almost every science, technology, business, management, organization, and industry [3, 23].

Machine learning (ML) is one of the most popular techniques used for the analysis of big data [3, 23]. ML is concerned about how computers can adapt to new circumstances, as well as detect and extrapolate patterns [4]. The essence of ML is that it is an automatic process of pattern recognition by a learning machine [23]. Machine learning mainly aims to build systems that can perform at or exceed human-level competence in handling many complex tasks or problems [4].

We are in the age of AI and big data. AI including NLP and big data have been applied to almost every sector and have been revolutionizing our work, lives and societies. AI is concerned with imitating, extending, augmenting, amplifying, and automating the intelligent behaviors of human beings [4]. AI attempts not only to understand how humans think, understand, write, learn, and act rationally and smartly but also to build intelligent entities that can think, write, perceive, understand, predict, and manipulate a world.

The relations among deep learning, machine learning, and AI are mathematically represented as follows: deep learning$\subset$machine learning $\subset$AI. In other words, deep learning is a subset of machine learning, and machine learning is a subset of artificial intelligence [4, 23].

Data mining is a process of discovering various models, summaries, derived values, and knowledge from a large database [24]. Data mining includes descriptive data mining and predictive data mining [24]. The former produces new nontrivial patterns and knowledge, while the latter produces models and roles of the systems. The primary tasks of descriptive data mining include clustering, summarization, and dependency modeling. The primary tasks of predictive data mining include classification, regression, change, and deviation detection [1, 4].

The relations among big data, data mining, and big data analytics are mathematically represented as follows: data mining$\subset$big data analytics$\subset$big data$\subset$data science. Data scientists aim to invent data intelligence-driven technologies and machines to represent, learn, simulate, reinforce, and transfer human-like intuition, imagination, curiosity, and creative thinking through human-data interaction and cooperation [17, 23].

NLP combines data mining with pattern associations to identify relationships between adjacent keywords in large big data sources [4]. For example, NLP may read languages other than English. NLP can understand human language in spoken or written format, by having the grammatical rules and comparing against those as words are processes from the big data. NLP may search any type of readable structured or unstructured data, including social media sites, job posting databases, or profiles stored anywhere on the internet. NLP makes judgments of phrase association strength based on distances between keywords in the same data and in the context of other words surrounding those of interest.

Enterprise resource planning (ERP) refers to an enterprise system that integrates business processes in manufacturing and production, finance and accounting, sales and marketing, and human resources into a single software system [1]. In other words, improving HR management with AI and big data has been an important part of ERP development [4, 28]. A big challenge is how to use AI, machine learning, and big data to improve HR management and the HR functions of ERP. The final goal of incorporating AI and big data analytics within HR management is to automate the recruitment processes and liberate the repetitive and time-consuming process of recruitment including interviewing a number of human applicants for any jobs. This research will contribute to the realization of this goal.

### Big data-derived small data approach

**Fig. 1:**
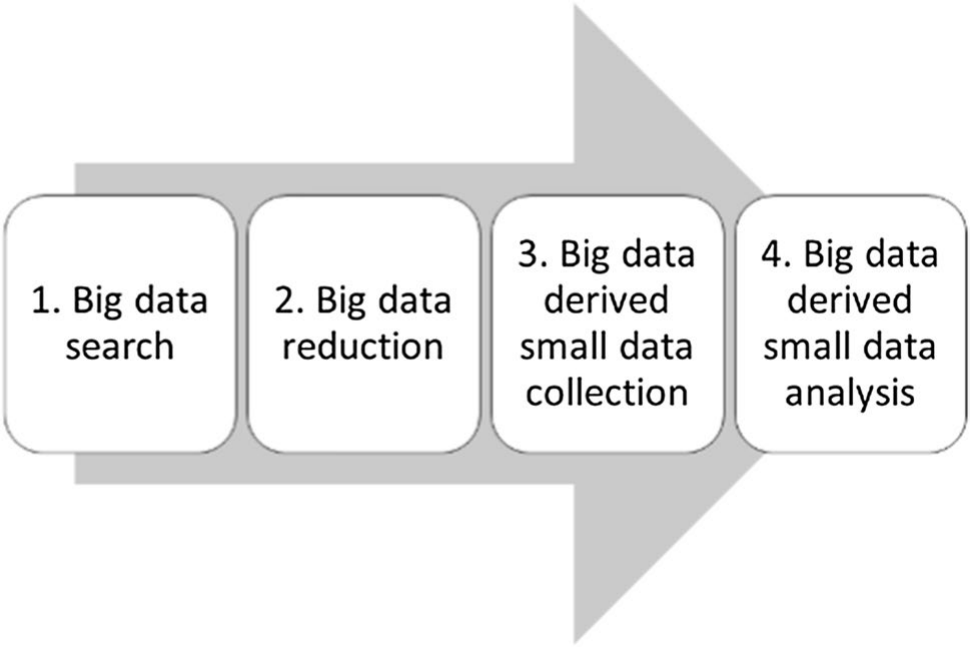
A big data-derived small data approach

Practitioners across all disciplines are living in the age of big data. However, sometimes, practitioners can only process small data analyses. This is a pragmatic contradiction. It may be a challenge to face the relativity between big data and small data. NLP can be applied to big data to derive a good fitting logical representation called small data. This subsection presents a dialectic unification between big data and small data by proposing a big data-derived small data approach based on [28]. As a process, as illustrated in Fig. 1, a big data derived small data approach consists of 1. Big data search; 2. Big data reduction; 3. Big data derived small data collection; and 4. Big data derived small data analysis. Big data search is the first step for the big data derived small data approach. Search all possible data, if not all existing data, is an important task for any research activity. It is also important for literature review, imagination, and association. In the big data search process, we can lead to the literature review, imagination, association, and revision of what we have planned and designed. This also implies that research as a search is the first step. In this step, we must identify where the big data resource for our research is.

In this research, we selected Google Scholar as the big data resource because it is the largest scholarly publication base in the digital world although EBSCO, Semantic Scholar, and Researchgate are good open-access (free) platforms for locating research. Therefore, we use Google Scholar to conduct big data searches.

Big data reduction is the second step for the big data derived small data approach. Reducing big data is, in essence, a kind of selection. The proper selection of data is to address how can we search for big data?

To address the second question, big data should be reduced in the big data search. That is, whenever searching, we must keep in mind that, big data reduction is critical for any big data search, otherwise, big data search would become the search of all the big data, for example, using Google for the entire Web. The heuristic method to address the second question is that we first analyze the research title and initial proposal or abstract of the research and obtain the important keywords or clauses in order to narrow the search space. For our search, we should apply the following keywords:

1. Big data human resources.
2. Artificial intelligence human resources.
3. NLP big data recruitment.
4. Artificial intelligence recruitment. Because artificial intelligence is usually abbreviated as AI, we should also select.
5. AI recruitment.
6. ERP human resources management.

This search has revolutionized our tradition that we usually relied on a few articles published in a few top journals and so-called important principles and results published in a few classic books or textbooks. The latter is equal to all the data searched from the big data search.

Big data derived small data collection is a special kind of sampling. Sampling can be applied to big data as a form of big data reduction. For example, Google Scholar should be a sampling, because Google Scholar cannot collect all the data of scholars on the Internet. There are two core parts for any sampling toward data analysis based on statistical inference. One is to collect what kind of data. The second is how to collect data. The former is related to what kind of data was important for the research. In other words, the importance of data is related to data analysis. The latter has been discussed in terms of statistical sampling. For the importance of data, we argue not all data need be taken for any decision-making and rule-seeking as well as statistical inference. Just as focusing on main problems with main solutions, one can also seek the most important data for any decision-making and statistical inference. Therefore, it is a big issue for every research to identify which data set is important to meet the objectives of the research.

For this research, what kind of data is important for this research to examine big data, AI, NLP, and recruiting? The possible answer is five types of data, that is, data on 1. Big data human resources, 2. Artificial intelligence human resources, 3. Big data recruitment, 4. Artificial intelligence recruitment. 5. AI recruitment” from Google Scholar (https://scholar.google.com/). Using this method, this research should collect up to 100 × 5 data results, that is, if we collect the data on the first 100 scholars’ publications in each area with the highest citation, then we can know the state-of-the-art big data, AI, NLP, and recruiting and their relationships.

We have still reduced the results using the criteria of citations if the search keywords or clauses are in the publication title or abstract. When we use a Google Scholar search for a keyword x, there are a lot of search results. This research collects about 100 × 5 data results. The 100 × 5 data items are small data, but it is derived from the big data of Google Scholar (http://scholar.google.com). Therefore, it is a big data-derived small data collection. This data collection is, in essence, a big data reduction for the proposed research.

Based on the above analysis, the corresponding searches were conducted on July 06, 2022. The search results are summarized in Table 1.

**Table 1:** Google Scholar search results and reference examples

	Keywords	Google scholar (m)	Examples
1	Big data human resources	88	V Koleva, L Mileva (2021) Opportunities for Big Data Application in Human Resources Management X Chen, L Yang, Y Sun (2021) Human Resource Information System Performance Test under Big Data Technology
2	Artificial intelligence human resources	101	BP Mariska, Y Prasetyo (2021) Perception and prospective analysis of artificial intelligence on human capital and its impact on human resources in the industrial revolution era 4.0 MAG Pillala (2021) Role of artificial intelligence in human resources
3	Big data recruitment	34	D Debao, M Yinxia, Z (2021) Min analysis of big data job requirements based on K-means text clustering in China
4	Artificial intelligence recruitment	189	J Wang (2021) Application of Keras neural network in the era of big data
5	AI recruitment	582	N Tilmes (2022) Disability, fairness, and algorithmic bias in AI recruitment. Ethics and Information Technology, Springer M Fritts, F Cabrera (2021) AI recruitment algorithms and the dehumanization problem. Ethics and Information Technology, Springer
6	ERP human resources management	46	JW Lee, HS Seo (2021) A Study on the AIT Gateway for human resources management modules extensions in ERP S Etehadnezhad (2021) Identifying the aspects and components impacting on enterprise resources planning (ERP) utilization with human resources empowerment approach in education

In Table 1, the second column consists of keywords for Google Scholar search. Google Scholar (m) in the third column represents the number of the Google Scholar search results. For example, a Google scholar search for “big data human resources” found 88 results. The examples in the fourth column listed two references with at least information on authors, year, and title, were searched for each corresponding keyword. The above search using Google Scholar demonstrates that.

1. Big data and Artificial intelligence have been used to improve human resources and human resources management.
2. ERP has been extended for improving human resources management.
3. We do not go into the deep literature review based on the selected 12 (= 6 × 2) literature in the context of big data derived small data approach, although they are useful for development of our research.
4. Only Google Scholar results for searching for “Artificial intelligence and AI recruitment” are much greater than 100.

Big data-derived small data approach is important both for the big data approach and big data analytics as a discipline [28]. First of all, big data has basically been controlled by a few global data giants such as Facebook, Google, Tencent, Baidu, and Alibaba rather than by an individual scholar. It is almost impossible for a scholar to use the big data of the mentioned giants to do research on big data-driven intelligent recruitment or similar. It is too expensive or unaffordable for a scholar to collect data and analyze the collected data because s/he has not a platform similar to that of the mentioned giant.

Secondly, most statistical inference based on sampling is reasoning based on incomplete knowledge or data [28]. Therefore, most statistical modeling or inference is a kind of big data derived small data analysis and reasoning [29]. This is also true for literature review in the age of big data. That is, the proposed big data-derived small data approach is a systemic method of the literature review in the age of big data.

Thirdly, from a data processing viewpoint, the largest data analysis could be performed in large data centers of a few global data monopolies running specialized software such as Hadoop over HDFS to harness thousands of cores to process data distributed throughout the cluster [29]. This means that individuals have to use big data derived small data analysis to analyze small but quality data.

Finally, any research in general and research publications, in particular, are, in essence, based on big data derived small data apporach, because an average research publication consists of 30 references, which has only up to 30 MB of data from a data volume viewpoint [17, 23]. In the big data world, the data with 30 MB is relatively small. In comparison, Amazon (AWS) and Google have processed 500 exabytes (EB) and 62 petabytes (PB) of big data in 2021 respectively [1, 2], where 1 EB = 1024 PB = 1024 × 1024 TB.

This research will use the above-proposed method to look at how AI and big data drive HR management in general and recruiting in particular in the next Sections.

### NLP AI and big data in ERP recruitment

In this subsection, NLP AI and big data problems are highlighted from the literature, particularly within the ERP talent recruitment function. The focus of this section is on empirical analysis of big data using AI. In other words, the preliminary literature RQ driving this section was: what actual AI techniques have been applied in ERP talent recruiting, and what were the key findings? This section is original in the literature because there are no other publications examining this preliminary literature RQ. In this section, the methodology is explained for conducting the literature review of AI and NLP in ERP using big data-derived small data analysis.

The first step in the literature review for this section was done using Google Scholar (https://scholar.google.com/). We searched “AI Human resource”, “artificial intelligence human resource”, “big data human resource”, “artificial Intelligence recruitment”, “NLP big data recruitment”, and “ERP HR recruiting AI” (see Table 1 and previous subsection). The notational relationships between these search terms are as follows:

Big data recruitment ⊆ big data human resource (HR);

ERP recruitment big data ⊆ big data human resources management (HRM);

Artificial intelligence recruitment ⊆ artificial Intelligence human resource (HR).

A Google Scholar search for “AI Human resource” (March 22, 2022) found 85 results. We first analyze the titles of the found results (based on the ranking of Google Scholar) in terms of “AI Human resource”. A Google Scholar (https://scholar.google.com.au/) search for “artificial intelligence human resource” found 95 results (retrieved on March 27, 2022).

It should be noted that only the first 70 results are related to the search keywords. The Google scholar’s search results are not exactly what we expected mathematically and algorithmically. For example, almost all the search engines on the web have not really realized the modus ponens, that is, $If A, \,A \to B, \,then B$. What Google or Google Scholar has provided is ${B}^{^{\prime}}, it$ satisfies$B \subset B^{\prime}$. We can call these as inclusion intelligence. Almost all the search engines use inclusion intelligence to provide the searched results to the users online. However, this is a weak intelligence from a mathematical viewpoint.

Compared with the search for “AI Human resource”, Google scholar’s search for “artificial intelligence human resource” found more results and more scholarly results. A Google Scholar (https://scholar.google.com.au/) search for “big data human resource” find 85 results (retrieved on March 27, 2022). Then we process these 85 results and delve into each of them. The analysis demonstrates that only the first 60 results are related to what we searched. Among them, there are also many results that are not really related to big data human resources.

Zang and Ye discussed how to apply big data to recruit talents for the enterprise [6]. They claimed that there are often prejudices in HRM recruiting. They asserted that big data can make recruiting fairer. This is because big data from the social networking platform can be viewed by peers and supervisors so candidates will likely be honest with their public profiles and resumes. Companies incorporate recruitment within online social networking services and constantly gather resume information and background information such as personal videos, personal pictures, living conditions, social relationships, and the self-reported competencies of the applicants. According to statistics, China has more than two-thirds of enterprises use online recruitment [25]. In fact, LinkedIn has been used as a part of recruiting talent.

Google Scholar, Semantic-scholar, LinkedIn, and Researchgate can provide big data on research activities, publications, and the background information of any scholar or job candidate [18, 26]. The information stored on the internet by those companies could be considered internet-based real-time big data. That big data is constantly changing, some of it is duplicated, and it is spread around the world on different servers. That data can be utilized in the ERP function of a company or university to identify potentially suitable candidates based on searches of mandatory hiring keywords. That big data may be evaluated and used to rank the candidates for a job opening. Interestingly, that same big data can also be evaluated to determine if the ERP talent recruitment process was fair, without race or skin color discrimination.

A Google Scholar (https://scholar.google.com.au/) search for “big data recruitment” find 31 results (retrieved on March 27, 2022). The algorithm in recruitment combining ML to assess big data sets in order to evaluate technical talent has been used in the recruitment practice [5, 10, 27]. Therefore, algorithmic recruitment is a kind of AI NLP and big data-driven recruitment. Evaluating big data to increase the efficiency and accuracy of recruitment and hiring decisions have drawn significant increasing attention [17]. Recently, a growing number of companies have used big data to build platforms and tools to help recruiters identify promising talents for jobs. Companies have also collected big data from a wide range of online social networking platforms such as Facebook, WeChat, and LinkedIn, where the potential candidate share their expertise from daily life to professional work [7]. Therefore, big data has made personal skills, knowledge, and performance transparent to the world. At the end of the day, big data-driven recruitment will become fair and fairer than the traditional one with obvious various discriminations because openness and global transparency can mitigate the prejudice and discrimination that often happened in the recruitment process.

A Google Scholar (https://scholar.google.com.au/) search for “artificial Intelligence recruitment” returned 163 results (retrieved on March 30, 2022). We analyzed the results based on the rank and order of Google Scholar. The preliminary analysis showed that AI recruitment has become an effective component of some firms’ entire promotion and human resource talent management processes [8]. For example, NLP AI techniques have been used in interviewing and assessment of candidates [2, 8, 10]. More generally, AI has been used to automate the recruitment process in human resources including tracking of applications, performance reviews, onboarding, compensation and career management, unbiased screening of candidates, scheduling, and decreasing of unconscious bias [2, 10].

ERP Human Resources Management (HRM or HR) is the management of people in a company or organization to achieve success in business performance. HRM is a strategic activity with the ERP function [1, 4, 10]. In the ERP function, HR departments are responsible for employee recruitment, overseeing employee-benefits design, training and development, performance appraisal, and reward management, such as managing pay and employee-benefits systems [16, 23]. Recruitment is a process of identifying, sourcing, screening, shortlisting, interviewing candidates, and offering the contracts for jobs (either permanent or temporary) provided by an organization. AI and big data have significant impacts on ERP in general and HRM recruitment in particular. For example, ERP was conceived to empower HRM to transform data into strategic knowledge and to develop talent through recruiting as well as training [1].

## Research approach

From a viewpoint of research methodology, this research uses a interdisciplinary approach consisting of the above proposed big data-derived small data approach, a pragmatic-driven ideology, practical analytical methods, computer tools, a big data-driven company case study site, and formal statistical techniques. This research used pragmatic-driven ideology, consisting of customized literature search and reduction techniques, and Microsoft Excel to collect and analyze the literature review.

The researchers held a pragmatic ideology, meaning that quantitative factual evidence was sought to prove deductive theories but practical approaches were designed to conduct a difficult case study. This can be called pragmatic-driven research design. The pragmatic ideology was applied because the researchers found it impossible to conduct a controlled experiment to compare a NLP AI algorithm with benchmarks when collecting internet-based job application real-time big data. Internet-based real-time big data cannot be stored in a single computer or a cloud database, at least not at the time of writing. Mixed sequential methods were applied, starting with machine learning at a case study site, followed by an evaluation by HRM staff at the case study company. Analysis of variance (ANOVA), Z-tests, and then Tukey post-hoc technique was applied to analyze the results in SPSS version 25. The authors developed two hypotheses based on the RQ and the literature review:

H1: Does the evaluation mean that the NLP AI program effectiveness is different from HRM staff? and if so, which ones were higher (could that be explained by staff error or an operational anomaly) ?

H2: Could ERP staff outperform the AI, by objectively selecting the best candidate from real-time big data using the same hiring criteria?

## Experiment

A case study company was selected. It is a government consulting firm in the IT sector based in the USA. The first author was the principal investigator (researchers). Approval was given by the researchers’ employers. The researchers conducted the data collection and quasi-experiment. Informed consent was obtained from the case study company, on the condition that everything except the data results would remain confidential. The case study company nominated one of its best-performing departments, the IT Security Development office (SDO). Project managers working in the SDO of the case study company were asked to update their resumes. Their resumes were parsed into fields and then assessed by nearest neighbor analysis in machine learning to identify the most important skill clusters. The most important clusters were used to build a job description with 21 mandatory criteria. These 21 hiring criteria became the evaluation scoring rubric to also be used by the HRM participants, as well as by the NLP AI software. An NLP AI -based recruiting program, CVViZ, was selected as the NLP AI software. The NLP AI recruiting module was written in JavaScript. It was compatible with the case study company’s legacy human resource information system (HRIS). Permission was given by the case study company to have their technology department install the NLP AI recruiting module into their HRIS for this experiment.

An ‘IT Development Manager’ job was created using the highest-ranked skills as criteria from the cluster analysis of the SDO staff resumes. The best resume was not used when prompted by the AI NLP software to avoid learning discriminatory behavior**s**. This was important to note because in earlier pilots loading the resumes produced skewed hiring criteria, due to the AI being trained to look for high frequencies of skills rather than important competencies needed for the job according to the strategic experience of the hiring manager.

Once the current study was started, it was time to connect the NLP AI application to the internet so real-time candidate profiles and application data could be searched. An internet search was activated to test the function and then all test data were deleted. In the experiment, the NLP AI software was used to screen candidates, and the application data were exported for the top candidate the AI had selected. The NLP AI search time from start to completion was captured into the HRIS database.

In parallel with the NLP AI program, human participants from the company were selected and trained to evaluate the candidate to be returned from the NLP AI. This human training was to ensure all HRM participants were familiar with the hiring criteria for the ERP specialized job (IT Development Manager), as well as how to use the scales to score the NLP AI. The training involved showing fictitious applications (profiles, cover letters, and resumes) of poor, medium, and good candidates. The evaluation scales were 1–10 for each criterion, aggregated, and then normalized to a score out of 100%.

Once the NLP AI program identified the best candidate from the internet real-time big data, the application materials were downloaded. The participants were individually given the application materials along with the evaluation rubric and asked to score the candidate on each hiring criterion as per the training given earlier. The scores from the 11 participants were downloaded and processed with ANOVA to answer the hypotheses. The time required for the NLP AI software to find the candidate was also downloaded and compared to the mean time HRM staff have taken in the last two years to find candidates for specialized positions. The organization benchmark was 8 h of effort. These data were processed with SPSS.

The researchers checked the accuracy of the exported candidate application data and compared the scores against hiring criteria to ensure the results were reasonable, with respect to avoiding discrimination. The result as interpreted by the researchers was reasonable—the candidate profile and resume indicated a well-qualified IT Development Manager and seemed to fit all hiring criteria very well.

## Results and discussion

The demographic characteristics of the 11 recruiters indicated all had at least 7 years of experience (M = 7.6, SD = 1.2), slightly more than half were female (55%), the average age was 38 (SD = 3.5) and all were employed in the IT staffing industry with a focus on IT project management. Most of the recruiters had a bachelor level education (52%), one had a Master’s degree, and the rest had Associate level degrees. The NLP AI software required 2 h to locate the specialized job candidate. The HRM staff took an average of 2.5 h to evaluate the NLP AI using the rubric. The benchmark time needed for HRM staff in the past to find a similar specialized job candidate was 8 h, using all HRIS tools (except the NLP AI software which had not been installed until this case study).

The first goal was to answer the RQ by testing the first hypothesis (H1): Does the evaluation mean that the NLP AI program effectiveness is different from HRM staff, and if so, which ones were higher (could that be explained by staff error or an operational anomaly)? The result was yes, and H1 was accepted. Table 2 contains the key estimates from the ANOVA technique, where evaluation score was the dependent variable, while the number of HRM staff was the fixed factor. The result was significant based on *F* (220, 10) = 18.1, *p* < 0.001 with an effect size of 44% (η^2^ = 0.44, Ѡ^2^ = 0.42).

**Table 2:** ANOVA estimates of comparison between recruiters and the AI recruiter

Cases	Sum of squares	DF	Mean squares	F	p		η^2^	η^2^_p_	ω^2^
Recruiter	51.185	0	5.724	18.1	< 0.001	5	0.44	0.44	0.42
Residuals	76.045	20	0.303						

Next, the second hypothesis (H2) was examined, could NLP AI algorithms outperform previous HRM staff effectiveness, by more quickly yet effectively selecting the best candidate from real-time big data? As discussed, prior to any statistical analysis, the researchers had reviewed the candidate returned by the NLP AI to ensure it was reasonable. The researchers looked at the evaluation scores on the rubrics by all HRM staff to confirm there were no missing fields and no outliers. The result was that the NLP AI software outperformed the previous HRM staff benchmark (Z[1, 10] = 18.1, p < 0.001) so H2 was accepted. These results will be analyzed in more detail below.

**Fig. 2:**
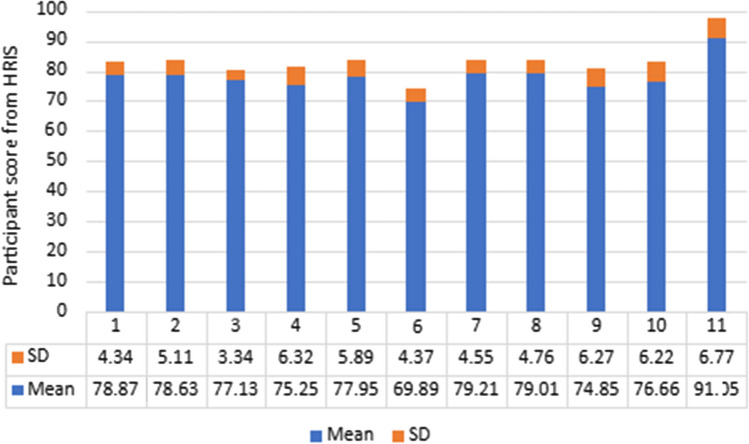
Job candidate evaluation scores of 10 recruiters and AI recruiter (11) using real-time big data

Figure 2 is a stacked bar chart illustrating the NLP AI evaluation scores calculated by the HRM staff using the rubric. In the rubric, each criterion was given a score out of 100 and these were averaged to form a total evaluation score, the mean evaluation score seen in Fig. 2 and Table 2. The y-axis was the mean evaluation score per participant out of 100%. The numbers on the x-axis represent the HRM staff participants conducting the NLP AI evaluation using the rubric of hiring criteria.

The descriptive estimates of the 11 HRM staff evaluation scores exported from the HRIS are listed in Table 3. In Table 3, it can be seen that HRM staff participant 11 had the highest candidate score (M = 91.05, SD = 6.77, refer to participant 11 in Table 3) which was significantly higher than any of the recruiters. Respondents 1, 2, 7, and 8 had candidate scores similar to one another but significantly lower than the NLP AI software, while recruiters 4, 5, 6, and 10 had lower scores and participant 3 had the lowest (M = 69.89, SD = 3.34).

**Table 3:** Descriptive statistics of evaluations

Participants	Mean	SD
1	78.87	4.34
2	78.63	5.11
3	77.13	3.34
4	75.25	6.32
5	77.95	5.89
6	69.89	4.37
7	79.21	4.55
8	79.01	4.76
9	74.85	6.27
10	76.66	6.22
11	91.05	6.77

Clearly the NLP AI was effective and performed faster than the HRM staff had performed previously (the organization benchmark), to select a specialized job candidate. Despite the researchers found that the evaluation scores were representative of the application data in each case, and the candidate application seemed reasonable, we do not know conclusively if the NLP AI software picked the best candidate from all internet application big data. That task would be daunting to prove. Nevertheless, we can conclude from this practical case study that the NLP AI software outperformed the HRM staff benchmark, and selected a good-fitting candidate for the specialized job position. These results answered the RQ and both hypotheses.

## Limitations and future research directions

Before anyone jumps to conclusions that this finding is generalizable everywhere, the limitations must be emphasized. This was a quasi-experiment, with very little control. Only the tool was controlled (the NLP AI software holding the job hiring criteria and selected applications). The sample size was small with 11 recruiters randomly selected on a social media platform using a poll feature that was closed in a day (to purposively limit too many respondents which would have caused the study to be unmanageable). There was minimal purposeful sampling because the respondents are self-selected. Since the applications were sourced from internet big data, we cannot know if during the 8-h time period over 1 day that some applications were deleted by candidates or maybe new ones would have been added. Thus, the data was not a point in time which the authors argue is impossible with big data (otherwise it is not really big data). Even so, this is a practice based on the principle of big data-driven small data analysis [28].

Another limitation is that we do not know what role pure chance or systematic error played, due to the quasi-experimental design without a control. For example, the time order of candidates applying might play a role since the AI recruiter was able to select better applications before those expired or were deleted from the internet. This was done without statistical replacement, so we do not know how well the AI recruiter would perform as compared to each of the 10 recruiters if everyone evaluated the exact same resume and cover letter. As noted earlier, it was not possible in order to satisfy the RQ, but it is certainly recommended in future research designs.

In summary, the current quasi-experiment was very limited. It was exploratory but with interesting and provocative results. Other researchers would need to replicate this and use alternative methods, possibly multiple case studies with embedded controlled experiments, to more fully compare the AI recruiter with experienced recruiters. Additionally, other recruiting AI software should be tested in a scientifically controlled manner such as in the current study.

## Conclusion

In the current study, a pragmatic quasi-experiment was used to answer the RQ. This was because the unit of analysis involved real-time big data, and actual candidate job applications sourced from the internet. It was not practical to download these, and using a sample would have defeated the goal to utilize actual big data. It was also not practical to control the case study as an experiment by selecting a resume or creating a fake one as done by other researchers [13–16]. Creating one resume and cover letter for experimental control would have canceled the realistic effect of analyzing real-time internet big data. The purpose was to force the NLP AI software to analyze actual relevant real-time big data.

We accepted both of our hypotheses, namely:

●H1: Does the evaluation mean that the NLP AI program effectiveness is different from HRM staff? and if so, which ones were higher (could that be explained by staff error or an operational anomaly) [supported by the data]; and,

●H2: Could NLP AI algorithms outperform previous HRM staff effectiveness, by more quickly yet effectively selecting the best candidate from real-time big data? [supported by the data].

We learned a valuable lesson that can be shared with the research community of practice, for the purposes of stimulating more studies. In the current case study, the NLP AI software outperformed previous HRM staff on the time, by a factor of more than 4 (2 h for the NLP AI versus 8 h for the ERP staff mean effort benchmark over the last two years). The NLP AI software selected a good-fitting candidate with a statistically significant high score of 90. This was a rigorous case study where the methods were explained in detail and the results were truthfully reported.

We might partially explain our results as falling into the Turing Test trap, a well-known phenomenon introduced by Alan Turing in 1950 [30]. In his experiment, computers outperformed human intelligence due to faster comparison algorithms where words, numbers, or other data were compared in tables. The point was even though computer-powered NLP AI may be faster than humans in certain comparative tasks, this does not necessarily mean that AI is always better than humans. Humans create AIs and humans are the ultimate decision-makers. This is still a very controversial topic in computer science, data science, cognitive science, and philosophy. This is the reason why we are studying AI with humans in this quasi-experiment.

The current study addressed other issues from the literature review. Some researchers proved that human recruiters have biases and they discriminate [8, 10, 27]. For example, human recruiters may consciously or subconsciously not select candidates who are of a certain gender or race, who did not attend an ‘ivy college’ in the USA nor 211 and 985 universities of China, who are too old, who are international, and even applicants who are not located in the city zip code of the posted job [14, 16]. The current study addressed those controversies to some extent, by listing hiring criteria identified by a relevant case study company, vetted by their HRM leaders, despite those skills being based on the resumes of their own high-performing staff. In the current study, the hiring criteria were worded to be discrimination-free, gender, and race-based criterion were not used, and no specific ivy school or accredited university in a certain country or state was mandated (or even mentioned). This forced the human recruiters (and the NLP AI software) to evaluate those hiring criteria and score against those, not a natural language learning algorithm to uncover personality clues from what or how candidates wrote their applications.

Furthermore, a few researchers found that recruiting NLP AI software could also discriminate, not prejudice in the same way as humans, but ML can make errors based on the ‘training’ procedure [9, 15, 16]. Remember, there was the Amazon ‘secret hiring AI’ used during 2014–2017, which rejected applications containing a woman’s salutation or name, and excluded certain colleges [9]. ML works by identifying patterns and looking for those. NLP AI software used in HRM generally includes ML and it usually prompts HRM staff to upload example best-in-class resumes, such as those already in the company. In the current study, this drawback was overcome because no best-in-class resume was uploaded into the hiring NLP AI program. Instead, which was a lot more work, hiring criteria were developed using experts at a relevant case study company, and those were input into the AI for selection criteria. Nonetheless, there was a BBC journalist who experimented with recruiting AI whereby she concluded women and people of color were being overlooked—including herself [12].

In the current study, NLP AI discrimination was prevented because the researchers made sure that the hiring criteria were not discriminatory in a way, including not citing a specific location or race/gender attributions. Finally, recall the MIT experiment to evaluate two hiring AI recruiters using a fake job, whereby both programs failed since among other errors a candidate scored high for English proficiency when she spoke only in German [13]. The current study avoided this problem because live interviews were not included in the quasi-experiment, since the purpose was to use the AI recruiter to select candidates from internet application big data. To carry this theme further, the authors would suggest the next step if this were an actual hiring event would be to interview all 11 candidates. Alternatively, the authors suggest the AI recruiter could be used to identify 5–10 best candidates, and then experienced human recruiters would take over and interview those, with humans making the complex final decision of the best candidate to hire.
